# Foreign Body Accidentally Lodged in the Larynx of a Child

**Published:** 1844-11-16

**Authors:** 


FOREIGN BODY ACCIDENTALLY LODGED IN THE LARYNX OF A CHILD.

Mr. R. W. Smith exhibited to the Dublin Pathological Society, the larynx of a child, six years of age, whose death was occasioned by a nail which it had in its mouth, and had accidentally swallowed; the nail, which was one of the common brass nails used by upholsterers in covering chairs, did not pass into the œsphagus, but got into the larynx, where it became fixed, and occasioned suffocation. The the child in this condition, was brought to the dispensary where every means for its relief were used, but in vain. It was at this time completely asphyxiated. Mr. Richey opened the trachea; a gush of air came from the lungs through the incision, but no inspiration followed, although inflation of the lungs was resorted to. The nail was found lying in the larynx, with its point upwards in the sinus of the ventricle of the larynx, and its head downwards below the glottis. Mr. Smith observed that we should not be deterred by the result of this case from attempting to restore animation in cases of asphyxis from similar causes: some cases were recorded, in which, after tracheotomy, or laryngotomy, the lung had been successfully inflated, even when no air had gushed from the wound.—*Dublin Journal.*

